# Structure of HIV-1 quasi-species as early indicator for switches of co-receptor tropism

**DOI:** 10.1186/1742-6405-7-41

**Published:** 2010-11-30

**Authors:** J Nikolaj Dybowski, Dominik Heider, Daniel Hoffmann

**Affiliations:** 1 Department of Bioinformatics, Center for Medical Biotechnology, University of Duisburg-Essen, Universitätsstr. 1-5, D-45117 Essen, Germany

## Abstract

Deep sequencing is able to generate a complete picture of the retroviral quasi-species in a patient. We demonstrate that the unprecedented power of deep sequencing in conjunction with computational data analysis has great potential for clinical diagnostics and basic research. Specifically, we analyzed longitudinal deep sequencing data from patients in a study with Vicriviroc, a drug that blocks the HIV-1 co-receptor CCR5. Sequences covered the V3-loop of gp120, known to be the main determinant of co-receptor tropism. First, we evaluated this data with a computational model for the interpretation of V3-sequences with respect to tropism, and we found complete agreement with results from phenotypic assays. Thus, the method could be applied in cases where phenotypic assays fail. Second, computational analysis led to the discovery of a characteristic pattern in the quasi-species that foreshadows switches of co-receptor tropism. This analysis could help to unravel the mechanism of tropism switches, and to predict these switches weeks to months before they can be detected by a phenotypic assay.

## Findings

Human Immunodeficiency Virus 1 (HIV-1) enters cells in a complex process involving interactions of viral envelope protein gp120 with the cellular receptor CD4 and a co-receptor, typically one of the chemokine receptors CCR5 or CXCR4 [1]. According to their co-receptor usage or "tropism", viruses are classified as "R5" (interacting with CCR5) or "X4" (interacting with CXCR4). Additionally, there are dual-tropic "R5X4" strains that use both co-receptors for cell entry. Tropism is mainly determined by the sequence of the variable loop 3 (V3) of gp120. In initial infection, R5 viruses dominate the viral quasi-species [2]. As the disease progresses, about 50% of the patients develop X4 virus [3]. CCR5 blocking drugs, such as Maraviroc or Vicriviroc [4,5] are ineffective against X4 virus, and thus it is advisable to test tropism prior to treatment with these drugs. The current state-of-the-art is testing by phenotypic assays such as Trofile^® ^(Monogram Biosciences, CA) [6] or enhanced sensitivity Trofile^® ^assay (ESTA) [7]. However, their restriction to specialized laboratories, high cost and long turn-around are limiting availability. Moreover, phenotypic assays have been reported to fail in delivering any result in more than 15% of the cases [8]. An alternative for routine diagnostics is genotypic testing: the genomic sequence of V3 from a patient is interpreted using computational models that relate V3 sequence and tropism. These models are typically derived by machine learning methods from a training set of V3 sequences and corresponding phenotypic test results [9-14]. Genotypic predictions can be made available via the Internet, and they are fast and cheap. Failure rates have been estimated to be around 7.5% [8]. In clinical settings with tropism predictions based on single sequences from bulk sequencing, genotypic methods tend to perform less well [15], which is mostly attributed to low detection rates of X4 minorities by bulk sequencing [16]. Genotypic testing based on so-called "next generation sequencing" or "deep sequencing" methods may not suffer from this limitation [17] as they provide detailed data for the whole viral quasi-species. In fact, Vandenbroucke *et al. *[18] have demonstrated that a combination of deep sequencing of V3 with a genotype interpretation algorithm [11,19] can be used for determination of tropism even in cases where phenotypic testing fails. In their study, the error rate of prediction methods was a limiting factor.

**Figure 1:**
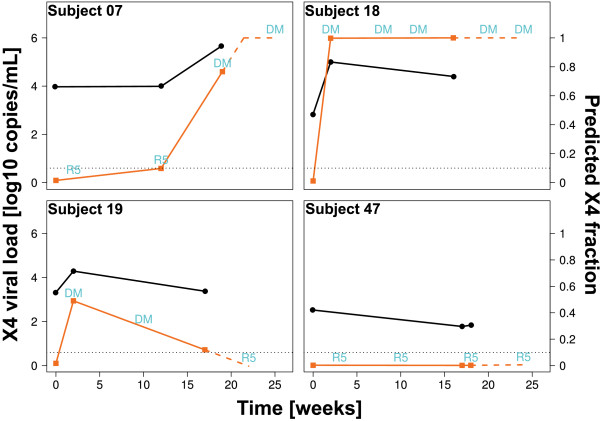
**Development of fraction of X4 viruses**. Development of predicted fraction of X4-using viruses during Vicriviroc treatment for four patients (right vertical axes, orange squares), and the contribution of that fraction to the absolute viral load (left vertical axes, black circles). Labels "R5" (R5-using) and "DM"(dual/mixed or X4-using) are Trofile^® ^results at the given times. The dotted line marks the 10% detection rate of standard Trofile^® ^assay. Naming was adopted from [21].

**Figure 2:**
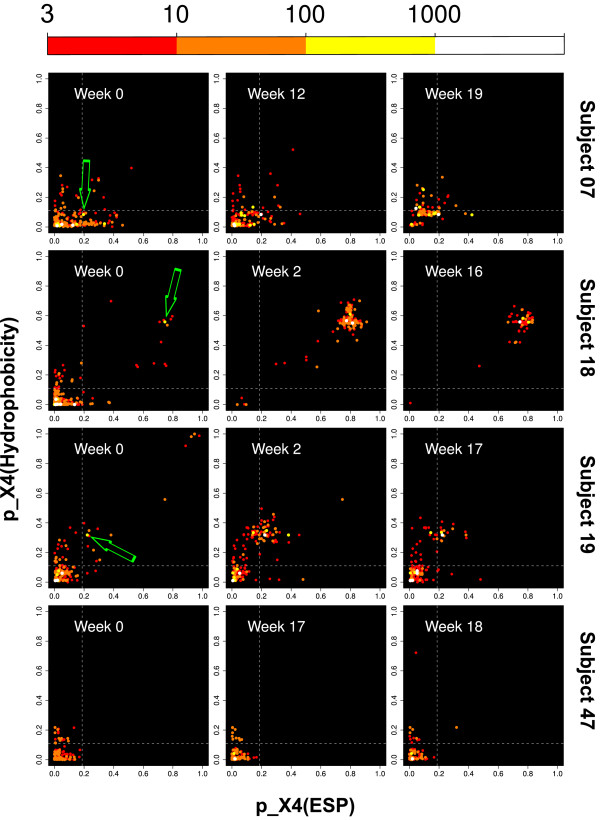
**Development of quasi-species**. Development of HIV-1 quasi-species in patients. Every dot represents a combination of two tropism predictions for the same V3 sequence: one prediction based on electrostatic potential (ESP), the other on hydropathy. Colors code the number of reads (color legend in top line). Dashed lines distinguish between R5 and X4 classes of the first level predictors. Cutoffs were chosen at 90% specificity in the training set. Arrows mark X4 seed strains responsible for tropism switch.

Recently, we have devised a two-level machine learning approach (T-CUP) [14] for the prediction of HIV-1 co-receptor usage from V3 sequences. At the first level, two independent predictions are made, based on the electrostatic potential and hydropathy values [20] of the V3 loop, respectively. The predictions are then combined, and a final decision is reached. The method is accurate, provides predictions for all subtypes and is robust with respect to insertions and deletions. In this study, we applied T-CUP to deep sequencing data from Tsibris *et al. *[21], compared the predictions with results of phenotypic assays, and analyzed features of the viral quasi-species that could indicate tropism switches. Tsibris *et al*. had generated deep sequencing data for four patients at three time points during treatment with Vicriviroc, and concurrently had measured phenotypic tropism with the standard Trofile^® ^assay. These assays had shown R5 virus at treatment start (Week 0 in Figure 1 and 2) for all patients. The common feature of all four patients was failure of Vicriviroc therapy.

We extracted V3 sequences in the viral quasi-species from the data published by Tsibris *et al*. by alignment to reference HXB2 V3 using Smith-Waterman local alignment [22] and translation into the corresponding amino acid sequence (Table 1). The tropism for every sequence in the quasi-species of each patient was predicted using T-CUP. The fraction of predicted X4 virus (i.e. number of predicted X4 tropic sequences divided by total number of sequences) in a quasi-species was compared with the outcome of the corresponding Trofile^® ^test. Figure 1 shows that there is a perfect agreement of the two Trofile^® ^classes R5 and DM (dual/mixed) with predicted X4 tropic fractions of below and above 0.1, respectively. This agreement holds for all four patients and all time points, and is concordant with reports of reliable detection of X4 minorities in test mixtures by the standard Trofile^® ^assay at concentrations down to 5-10% [6]. The phenotypic tropism assay has dichotomous output (either R5 or DM), while the T-CUP analysis of deep sequencing data generates a practically continuous (fraction of X4 tropic virus in quasi-species in units of 1/(number of reads)). The latter allows for a more detailed characterization of the dynamics of the quasi-species with respect to tropism. It should be noted that the density of sampling points along the time axis in the Tsibris *et al*. dataset is too small for an accurate modeling of the tropism dynamics. However, the slopes of the lines in Figure 1 illustrate the principle. For instance, in the computational analysis of the quasi-species in Subject 07 we see an increase of the X4 fraction from week 0 over week 12 to week 19. From the slope between week 0 and week 12 we could extrapolate that shortly after week 12 a switch from R5 to DM should occur in the phenotypic assay. In fact, the phenotype data shows that the tropism switches between weeks 12 and 19 from R5 to DM. In the same way we would expect that the virus in Subject 18 remains DM tropic, and in Subject 47 R5 tropic. Subject 19 is a particularly interesting case with an early switch from R5 to DM, accompanied by a steep increase in the X4 fraction according to T-CUP to 0.5 at week 2. Then the X4 fraction drops to slightly above 0.1 at week 17. From this development we could extrapolate a reversion from DM to R5 shortly after week 17 (dashed blue line in Figure 1), as observed by Tsibris *et al. *[21].

**Table 1:** Unique V3 sequences

Patient	time 1	time 2	time 3
07	174	112	86
18	240	112	41
19	148	134	104
47	126	84	78

Number of unique sequences used in the analysis for each patient and each of the three sample times. Based on the data provided by Tsibris *et al*., a cut-off of a minimum of 4 reads per sequence was applied to limit the number of spurious sequences.

We next exploited the property of T-CUP to provide in the first level two independent tropism predictions based on physical properties (electrostatics and hydropathy) of V3. The corresponding probabilities span a plane ("probabilities plane") in which every V3 sequence is represented by a point and the quasi-species by a cloud of such points. Figure 2 shows this plane for all twelve datasets from Ref. [21] with the points colored according to frequency of the respective sequence in the deep sequencing data.

The dynamics of the quasi-species in the probabilities plane has several remarkable features. First, all sequences in week 0 cluster in the lower left corner of the plane as is expected for a quasi-species that is R5 tropic. Second, the movement of the clouds indicates the dynamics of tropism. For Subjects 07 and 18 the clouds move towards the upper right, i.e. to more X4 tropism. For Subject 19 this movement is also seen for the first two time points but then reverts again to the lower left, i.e. to more R5 tropism. Subject 47 shows no marked movement to the upper right but remains localized in the lower left, in agreement with a quasi-species that remains R5 tropic. Third, for the patients 07, 18, and 19 where a co-receptor switch had been observed, there is only one clearly dominating X4 strain in the probabilities plane, and this strain is already present at therapy start with considerable frequency (bright spots with green arrows). This "X4 seed strain" is specific for each of the patients - the seed strains for different patients are clearly located in different regions of the probabilities plane. Additionally, the X4 seed strain is accompanied from the beginning by a growing halo of local minor variants. Note that Subject 47 who remains R5 tropic throughout all time points does not have such a cluster.

**Figure 3:**
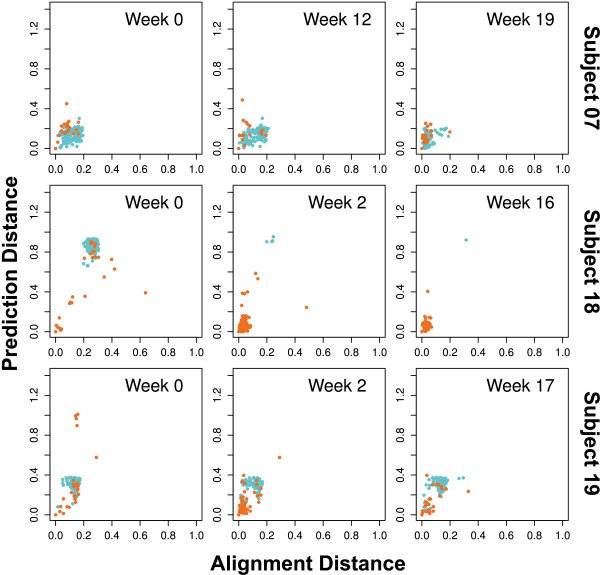
**Homology to X4 seed strain**. For each sequence *s*_i _the Euclidean distance to the seed sequence *s_seed _*in the probabilities plane (Figure 2) is given on the vertical axis, while the horizontal axis shows the "alignment distance" to the seed sequence given by (1 - *A*(*s*_i_, *s_seed_*))*/*(*A*(*s_seed_*, *s_seed_*)) with sequence alignment score *A *based on Needleman-Wunsch alignment [23] with scoring matrix BLOSUM62 [24]. The lower the alignment distance, the higher the homology to the seed strain. Sequences predicted as "X4" are orange, those predicted "R5" cyan. The seed sequence is the red point in lower left corner. The figure shows that sequences close to the seed strain in Figure 2 have also low alignment distance, i.e. are close homologs.

We interpret the seed strain with its halo as a variant that has established itself in the quasi-species even at week 0 so that it can generate a considerable number of copies and also generates variant offspring. This interpretation is supported by the high homology of the sequences in the cluster (Figure 3). Using the statistical properties of this cluster in the probability plane (strong seed strain, halo of neighbor strains), it may become possible to *predict *a future co-receptor switch and therapy failure many weeks earlier than the switch of tropism becomes manifest in a phenotypic test. For such an early detection of a later switch of tropism, the resolution of deep sequencing and the accuracy of the prediction method is critical. For all three subjects where a switch occurs, the X4 seed strain initially accounts for 0.5% to 1% of the quasi-species (Table 2). The following progression towards X4 tropism is almost exclusively due to the expansion of these seed strains. For the three patients with a tropism switch a linear correlation of development of the total X4 fraction over time with the development of the fraction of the seed strain over time yields *R*^2 ^= 0.98 (*p *= 7.7 · 10^-10^).

**Table 2:** Development of X4 variants causing tropism switch

Patient	Variant	Fraction of population at		
		time 1	time 2	time 3
07	CTRPGNNTRRSIRIGPGQTFFAREDIIGDIRQAYC	0.01	0.07	0.73
18	CERPNNNTRQRLSIGPGRSFYTSRRIIGDVKKAHC	0.005	0.79	0.71
19	CTRPNNNTRKGIYLGPGRAFYTTDKIIGDIRQAHC	0.007	0.43	0.08
47	CTRPNNSTRKSINIGPGSAWYTTGDIIGDIRQAHC	0.0009	0.0	0.0

Development of the X4 seed strains in Subjects 07, 18, 19 with tropism switches and the largest initial X4 strain in patient 47 who does not show a tropism switch. The three times are the sampling points along the "Time" axis in Figure 1.

Although the high cost of deep sequencing will probably prevent its use in routine diagnostics in the near future, the combination of this powerful method with accurate predictions could be applied when phenotype testing fails and to study evolution of viral quasi-species under selective pressure, and thus contribute to the development of sustainably effective treatments.

## Competing interests

The authors declare that they have no competing interests.

## Authors' contributions

JND devised and carried out the research, analyzed data, and drafted the manuscript. D Heider contributed to data analysis and to drafting of the manuscript. D Hoffmann has devised research, analyzed data, and revised the manuscript. All authors read and approved the final manuscript.
